# A space-time analysis of mortality in older people living with HIV/AIDS in the state of São Paulo, Brazil

**DOI:** 10.1590/1980-549720230035

**Published:** 2023-07-28

**Authors:** Katyucia Oliveira Crispim de Souza, Ana Clara Cintra Santana, Vinícius do Nascimento Alves, Caíque Jordan Nunes Ribeiro, Allan Dantas dos Santos, Anna Luiza de Fátima Pinho Lins Gryschek

**Affiliations:** I Universidade de São Paulo, School of Nursing – São Paulo (SP), Brazil.; II Universidade Federal de Sergipe, Department of Nursing – Lagarto (SE), Brazil.

**Keywords:** Elderly, Space-time analysis, HIV, Acquired immunodeficiency syndrome, Idoso, Análise espaçotemporal, HIV, Síndrome da imunodeficiência adquirida

## Abstract

**Objective:**

To analyze the spatiotemporal distribution of mortality in older people living with the human immunodeficiency virus/acquired immunodeficiency syndrome (HIV/AIDS) in the state of São Paulo, Brazil.

**Methods:**

This is an ecological study with temporal and spatial approaches to analyze mortality from HIV/AIDS in the older adult population in the 2010-2020 period in the state of São Paulo, Brazil. Analysis of temporal trends was performed using the joinpoint regression, and spatial analysis was carried out using the Moran's index and the local empirical Bayesian model.

**Results:**

We identified a total of 3,070 deaths from HIV/AIDS among older adults and a mortality rate of 51.71 per 100 thousand inhabitants during the study period. The joinpoint method showed a growing trend for the age groups from 70 to 79 years (annual percent change [APC]=3.45; p=0.01) and ≥80 years (APC=6.60; p=0.006) and stability for the general older adult population (APC=0.99; p=0.226). The spatial distribution of the crude mortality rate was diffuse throughout the state. After smoothing by the Bayesian estimator, we observed greater concentration in the eastern mesoregions. In Moran's analysis, we observed clusters of lower mortality rates in more central regions; and of higher rates in the southern and northern regions of the state.

**Conclusions:**

We found a major growing trend in mortality from HIV/AIDS in the age group of older adults over 69 years during the 2010-2020 period. Clusters of high mortality rates were located in regions further to the south and north of the state, where places of greater social inequalities are concentrated.

## RESUMO

**Objetivo:**

O estudo tem como objetivo analisar a distribuição espaço-temporal da mortalidade em idosos que vivem com HIV/AIDS no estado de São Paulo, Brasil.

**Métodos:**

Estudo ecológico com abordagens temporal e espacial para análise da mortalidade por HIV/AIDS em pessoas idosas no período de 2010–2020 no estado de São Paulo, Brasil. A análise das tendências temporais foi realizada por meio da regressão joinpoint e as análises espaciais foram realizadas usando o índice de Moran e o modelo bayesiano empírico local.

**Resultados:**

Foram identificados 3.070 óbitos por HIV/AIDS entre pessoas idosas e taxa de mortalidade de 51,71 por 100 mil habitantes no período de estudo. O método *joinpoint* revelou tendência crescente para as faixas etárias de 70 a 79 anos (variação percentual anual — APC=3,45 p=0,01) e 80 anos ou mais (APC=6,60, p=0,006) e de estabilidade para a população idosa geral (APC=0,99, p=0,226). A distribuição espacial da taxa bruta de mortalidade demonstrou-se difusa em todo o estado. Após suavização pelo estimador bayesiano, observou-se maior concentração nas mesorregiões ao leste. Na análise de Moran, foram observados aglomerados das menores taxas de mortalidade em regiões mais centrais e das altas taxas em regiões mais ao sul e norte do estado.

**Conclusão:**

O grupo etário em que ocorreu maior tendência de crescimento da mortalidade por HIV/AIDS durante o período de 2010–2020 foi o de pessoas idosas com mais de 69 anos. Os aglomerados das altas taxas de mortalidade foram localizados em regiões mais ao sul e norte do estado, onde se concentram locais de maiores desigualdades sociais.

## INTRODUCTION

The acquired immunodeficiency syndrome (AIDS) is an important public health issue, due to its global nature, costs with health care, prevention, injuries resulting from lack of treatment for people living with the human immunodeficiency virus [HIV]/AIDS (PLWHA), and the stigma that still exists about the disease^1^. During the initial years of the HIV/AIDS epidemic, there were few treatment options. However, with the advancement of scientific knowledge of the infection and the development of new medications, which compose antiretroviral therapies (ART), as well as public policies for testing and treatment, a decrease in morbidity and mortality due to the disease and a change in the natural course of the virus were observed^2^.

HIV/AIDS has become a chronic disease, and there has been a drastic reduction in mortality compared with the initial years of the epidemic^3^. During the period from 2010 to 2019, there was a 39% decrease in deaths globally, but mortality numbers are still high, with 690 thousand deaths from AIDS recorded worldwide in 2019^4^.

In Brazil, an average of 41,1 thousand new cases of HIV/AIDS are diagnosed every year, 40% of which are identified late, symptomatic, or asymptomatic with a CD4+ lymphocyte count <200 cells/mm^3,5^. Mortality from the disease in the country has shown high numbers — around 5.3/100 thousand inhabitants in the period from 2008 to 2018 — and has remained stable^6^.

The Brazilian Southern and Southeastern regions concentrate most of the diagnosed cases, with emphasis on the state of São Paulo (SSP), where there was a 19.6% increase in the total number of cases of HIV infection in the last ten years, from 5,295 cases in 2010 to 6,332 in 2020. Epidemiological analysis shows that, between 2010 and 2019, SSP registered 34,480 deaths from the disease, and mortality rates have been decreasing since the introduction of ART, following the global trend^7^.

The mortality rate in SSP decreased from 7.6 deaths per 100 thousand inhabitants/year in 2010 to 4.6 deaths per 100 thousand inhabitants/year in 2019. Nevertheless, the number is worrisome, considering that the reduction in the number of deaths is spatially heterogeneous, with regions with 2.0 deaths per 100 thousand inhabitants/year, such as the cities of Presidente Prudente and Registro, and others with 7.5 deaths per 100 thousand inhabitants/year such as the municipality of Santos^7^.

Even with a decreasing trend of new cases, some population groups are worrisome for not following this pattern. This is the case of older adults, as the accelerated process of population aging has caused profound changes in the epidemiological profile of HIV/AIDS. In Brazil, in 2007, 643 deaths were reported in people aged over 60 years (5.65% of the total of 11,372); in 2016, there were 1,389 deaths of older people due to AIDS, representing 11.08% of the total of 12,540 deaths from the disease in that year^8^.

Studies demonstrate that there are many variants that boost the number of cases among older adults. With the increase in life expectancy, an increase in the sexually active period is also expected, which sometimes occurs in an unprotected way due to ingrained habits, as a large part of this group is composed of widowed and divorced individuals. In addition, the perception of the risks of the disease by older adults is lower, considering that the guidelines on sexually transmitted infections (STIs) are more deficient due to the existing taboo about sex in older people^9–11^.

In this context, further studies are needed regarding the spread of HIV/AIDS among older people and its consequences due to the vulnerability of this group. The use of techniques to identify geographic areas and affected populations over time is an effective strategy for a better analysis of the context and policies aimed at HIV/AIDS. Thus, the objective of this study was to analyze mortality trends and the spatial distribution of mortality from HIV/AIDS among older people from SSP, between the years 2010 and 2020.

## METHODS

### Study design

This is an ecological, descriptive, and population-based study, which used secondary data on deaths from HIV/AIDS among older people registered in SSP during the 2010–2020 period.

### Study location

**Figure 1:**
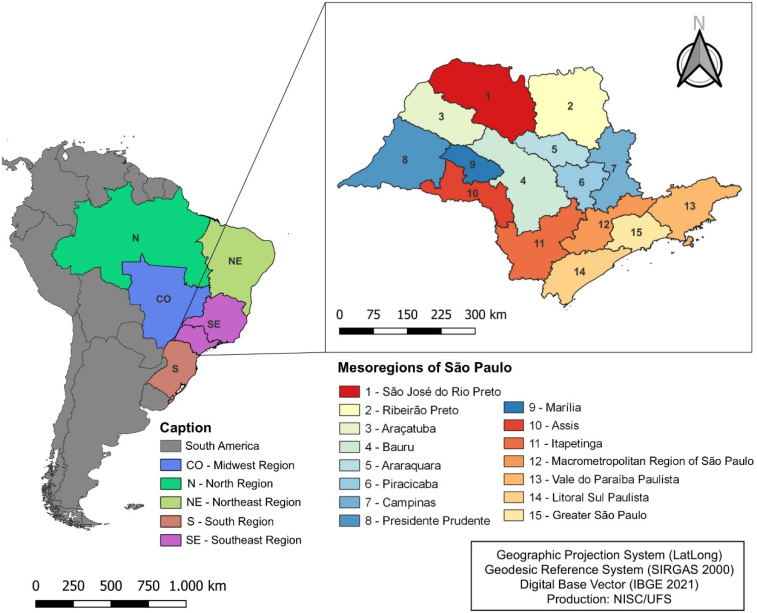
Mesoregions of the state of São Paulo, Brazil.

The analysis units were the 645 municipalities that are geographically distributed in the 15 SSP mesoregions (Figure 1), located in the Southeast Region of Brazil. Extending from the coast to the countryside, it is located at longitude 49 West and latitude 22 South, with a time zone of −3 hours in relation to world time GMT. São Paulo has a total area of 248,209.426 km^2^ and an estimated population of 46 million people. It is the most populous federation unit in Brazil, with 22% of the national population, and also the richest, with a gross domestic product of USD 603.4 billion^12^.

### Data collection

Data were collected from the Brazilian Mortality Information System (*Sistema de Informações sobre Mortalidade* – SIM), from the Department of Informatics of the Brazilian Unified Health System (*Departamento de Informática do Sistema Único de Saúde* – DATASUS) and from information on the studied population obtained from the Brazilian Institute of Geography and Statistics (IBGE). All deaths from HIV/AIDS in older people in the period from 2010 to 2020 were included, considering that the deaths correspond, according to the International Classification of Diseases (ICD), to codes B20 to B24 (ICD-10). Population data were obtained from the IBGE website.

### Data analysis

Annual mortality rates from HIV/AIDS among older people in the municipalities were estimated for 100 thousand inhabitants, using the corresponding annual population as the denominator. The mean crude mortality rate from HIV/AIDS in older adults in the period was estimated using the total number of deaths as the numerator and the population for the year 2015 as the denominator, which was later multiplied by the constant 100 thousand. Lastly, the local empirical Bayesian estimator was used to minimize the instability caused by the random fluctuation of cases, smoothing the standardized rates by applying weighted means, and creating a third corrected rate^13^. For the characterization of the study population, the epidemiological variables sex, education, marital status, age group, ethnicity/skin color, and area of residence were used, described in absolute and relative frequencies.

### Temporal analysis

The temporal trend analysis was performed using segmented linear regression, performed in the Joinpoint software v. 4.2.0. The HIV/AIDS mortality rate in older people was considered the dependent variable and the year of occurrence, the independent variable. The Monte Carlo permutation test was used to select the best segment of the model, considering the best model the one with the highest residual coefficient of determination (R2). To describe and quantify the trend, the annual percent change (APC) and its respective 95% confidence interval (95%CI) were estimated, considering statistical significance if the APC presented p<0.05 and its 95%CI did not include the value of zero. A positive and significant APC value indicates an increasing trend; if negative and significant, a decreasing trend. Conversely, nonsignificant trends are described as stable, regardless of APC values^14,15^.

### Spatial analysis

The calculation of the univariate global Moran's index was used to verify whether the spatial distribution of the phenomenon occurs randomly in space. This index ranges from −1 to +1, in such a way that values close to zero indicate spatial randomness; values between 0 and +1 indicate positive spatial autocorrelation; and between −1 and 0, negative spatial autocorrelation. The identification of regions with significant spatial correlation was verified by the local Moran's index (Local Index of Spatial Association *–* LISA), which compares the value of each municipality with neighboring municipalities, verifying the spatial dependence^16^.

Thus, a scatter plot was obtained with the following spatial quadrants: Q1 (high-high) and Q2 (low-low), which indicate municipalities with values similar to those of their neighbors and represent areas of agreement with clusters of positive spatial association; and Q3 (high-low) and Q4 (low-high), with different values, which represent transition areas with clusters of negative spatial association. Significant results were visually expressed on Moran's maps^16^.

TerraView version 4.2.0, QGis version 3.4.5, and GeoDa™ 1.14 programs were used to draw the maps using the cartographic base, in shapefile format, in the latitude/longitude geographic projection system (Geodetic Reference System for the Americas — SIRGAS 2000) of SSP, available from the IBGE website.

### Ethical aspects

The study followed the ethical standards of Resolution 466/12 of the National Council for Ethics in Research involving Human Beings and was approved by the Ethics and Research Committee of the School of Nursing of Universidade de São Paulo (Certificate of Presentation for Ethical Consideration — CAAE 56585622.1. 0000.5392 and Opinion No. 5.326.296).

## RESULTS

We identified a total of 3,070 deaths from HIV/AIDS in older adults, with a mortality rate of 51.71 per 100 thousand inhabitants in the period from 2010 to 2020, in SSP. There was a predominance of men (65.7%), aged between 60 and 69 years (71.9%), single (29%), white (64.60%), and with low level of education (maximum of three years of formal education) (22.3%) (Table 1).

**Table 1:** Sociodemographic variables of HIV/AIDS deaths in older people from 2010 to 2020, São Paulo, Brazil.

Variable		Deaths from HIV/AIDS n %	
Sex			
	Men	2,018	65.7
	Women	1,052	34.3
Age group			
	60 to 69 years	2,206	71.9
	70 to 79 years	686	22.3
	≥80 years	178	5.8
Ethnicity			
	White	1,983	64.6
	Nonwhite	988	32.2
	Ignored	99	3.2
Marital status			
	Single	889	29.0
	Married	784	25.5
	Widowed	630	20.5
	Divorced	530	17.3
	Other	65	2.1
	Ignored	172	5.6
Level of education			
	None	218	7.1
	1 to 3 years of formal education	684	22.3
	4 to 11 years of formal education	1,174	38.3
	≥12 years of formal education	222	7.2
	Ignored	772	25.1
	Total	3,070	100

**Figure 2:**
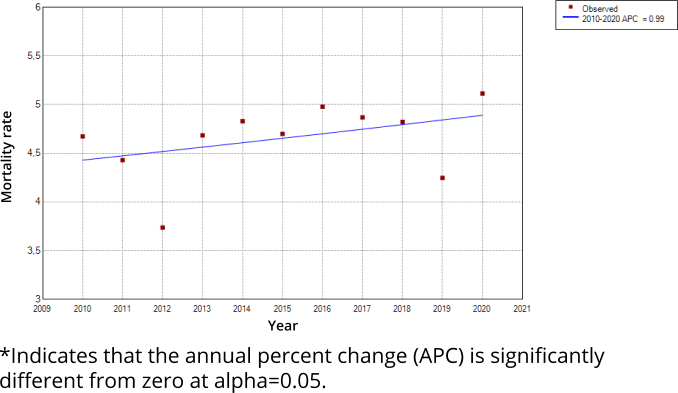
Temporal trend of HIV/AIDS deaths in older people from 2010 to 2020 in the state of São Paulo, Brazil.

Regarding the temporal trend analysis, the number of deaths among older people remains stable, with APC=0.99 (95%CI and p=0.226), which indicates a nonsignificant increase trend (Figure 2).

**Figure 3:**
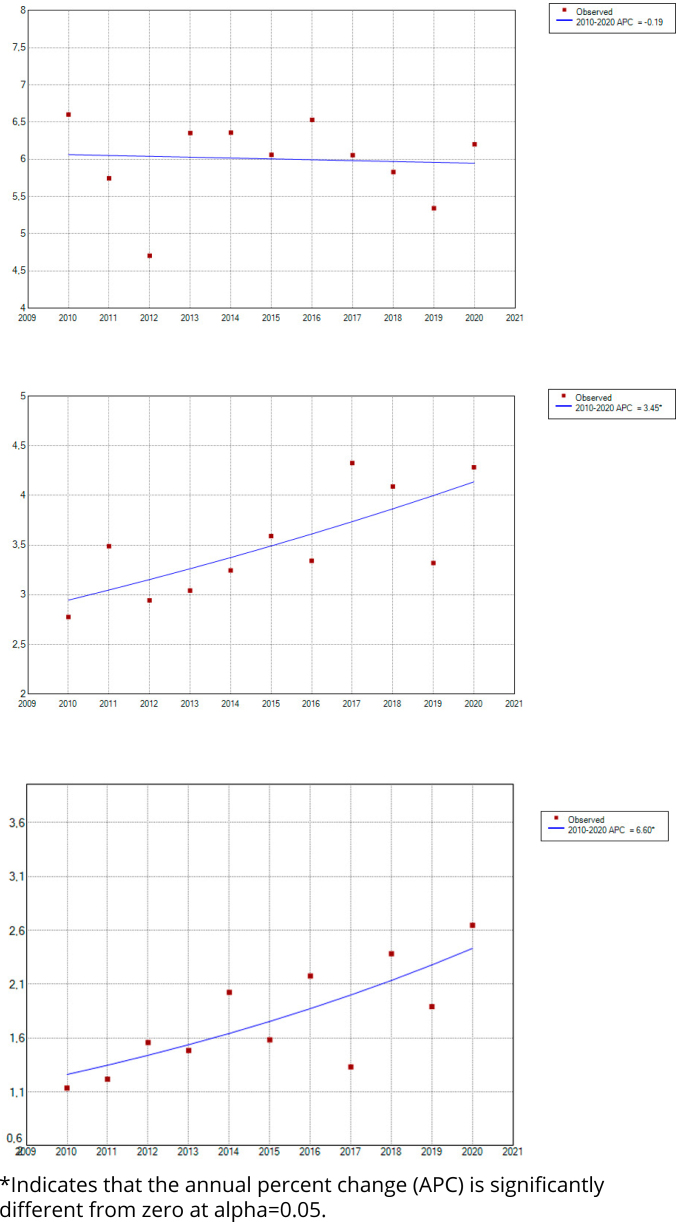
Temporal trend of deaths from HIV/AIDS in the age groups of 60 to 69 years, 70 to 79 years, and 80 years or over, from 2010 to 2020, in the state of São Paulo, Brazil.

As for the analysis by sex, men presented APC=0.70 (95%CI and p=0.418) and women, APC=1.56 (95%CI and p=0.227). The age group that showed the greatest growth in the period was 80 years or over, with APC=6.60 and p=0.006, followed by older adults aged 70 to 79 years, with APC=3.45 and p=0.010. As for the age group of 60 to 69 years, we observed no significant trend in the period (APC=0.19 and p=0.838) (Figure 3).

**Figure 4:**
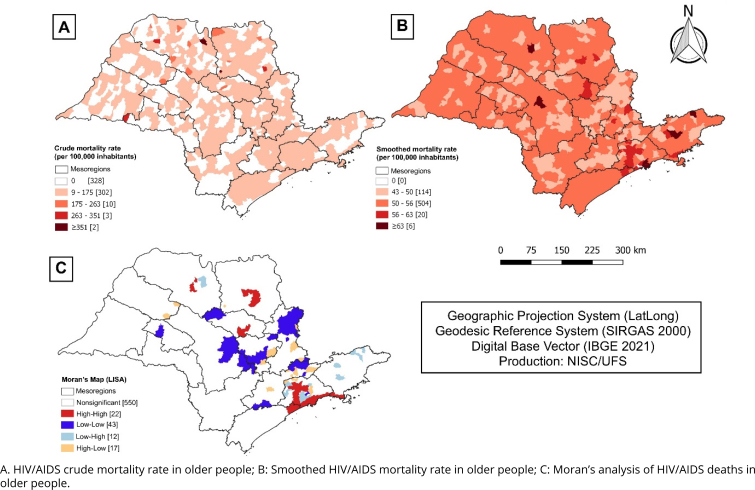
Spatial analysis of deaths from HIV/AIDS, from 2010 to 2020, in the state of São Paulo, Brazil.

The spatial distribution of the crude mortality rate due to HIV/AIDS in older adults was diffuse throughout the state, although it was more intense in the northern mesoregions of the state (Figure 4A). After smoothing by the Bayesian estimator, we observed greater concentration in the eastern mesoregions of the state (Figure 4B).

The global spatial autocorrelation analysis showed that mortality is heterogeneously distributed in space (I=0.219 and p=0.001). The analysis of the Moran's map allowed us to identified areas classified according to the significance level of their local indices. We identified clusters in the following mesoregions: São José do Rio Preto, Ribeirão Preto, southern Araraquara, Vale do Paraíba Paulista, Greater São Paulo, and Litoral Sul Paulista, with high mortality rates (Q1: high-high), and clusters with low rates in the mesoregions of Marília, northern Araraquara, Campinas, Bauru, Piracicaba, and Macrometropolitan Region of São Paulo (Q2: low-low) (Figure 4C).

## DISCUSSION

An accelerated aging process is taking place in several countries, bringing not only changes in the epidemiological profile, but also cultural and social transformations. Such changes in the lifestyle and work habits of the older adult population contributed to changing the way these people and their sexuality are perceived. Factors, such as social inclusion, the establishment of new sexual and affective bonds, and their consequences for the health of this population, brought about the need for further discussions on the subject^17^.

For a long time, the idea that sexual decline was inevitable in old age and that it should be accepted as something normal in the aging process. However, that is currently a consensus on aging as something complex, heterogeneous, and which does not represent a synonym of functional incapacity, dependence, or absence of social and sexual experiences^18^.

Authors of a study carried out at a gynecology outpatient clinic in the state of Espírito Santo pointed out that, according to the perception of a group of older people interviewed, the physical and emotional satisfaction promoted by sexual intercourse significantly contributes to psychological well-being and quality of life of this population^19^. In this sense, in addition to meeting a physiological need of individuals, sexuality positively contributes to the quality of life of older adults, aiming at well-being, self-esteem, and the search for an intimate relationship with another person. Nonetheless, when sexual activity is associated with unsafe practices, the vulnerability of this population to STIs, such as HIV/AIDS, increases^17^.

Researchers of a study carried out by Universidade Federal de São Paulo (UNIFESP) pointed out that HIV/AIDS is more prevalent in men, as this group has low adherence to condom use for several reasons, including difficulty using it, feeling of decreased erection and loss of sensitivity during the sexual intercourse, belief that there is no need to use it in affective or monogamous relationships, or because they believe that the pause to put on the condom “ruins the mood” of the intercourse^18^. In the present study, we also observed that men were most prevalent among cases of deaths from HIV/AIDS in older adults in the state of São Paulo.

In addition, we observed that the number of older women infected by the disease has increased. The causes for this increase are closely linked to patriarchy, which for centuries placed men in a position of superiority over women, resulting in female submission to the partner's wishes during sexual life^20^. Authors of a study carried out in a small city in the state of São Paulo concluded that older women are more prone to STIs because they do not find it necessary to use condoms when they are in long-term affective relationships, such as marriages and common-law marriages; or when reaching the climacteric^21^.

As observed in this study, most of those infected were older adults with less opportunity to access formal education. According to Hogan et al., low level of education is one of the social determinants that affects knowledge of the risk of the disease, the status of its treatment, and the risk of death — in addition to interfering with the understanding of health education programs, especially for older adults who have sociocultural limitations of access to current technologies^22^.

Sexuality among older people is considered nonexistent by the general population. This prejudice, which health professionals themselves often have, becomes another barrier to the effectiveness of sex education among these individuals^23^. The analysis also demonstrates that the most affected group is single and divorced older people, which corroborates findings described in the literature, as this group usually has more than one sexual partner^9^. These data show that it is increasingly urgent to implement sexual health programs aimed at older adults to reduce the incidence of STIs and, consequently, mortality in this population.

We noticed that the disease affects several people indiscriminately, regardless of age, sex, or sexual orientation. According to Castro et al.,^1^ there was an increase in the incidence of HIV/AIDS in the state of Minas Gerais in all age groups, including older people. In the states of Piauí and Ceará, there was a considerable increase in cases, especially in older adults^24,25^.

The increasing number of deaths from HIV/AIDS in older people aged 70 years or over, observed in the temporal analysis, is alarming and can be explained by the high rate of infection associated with low testing in this specific population. The lack of sexual health promotion campaigns and the taboo on sexuality in older adults are related to the underuse of tests, which can lead older people not to seek guidance on safe sexual practices or adequate treatment^26^.

Conversely, we can associate this issue with advances and greater accessibility to HIV treatment, which has reduced early mortality from the disease, and the affected people are able to have a better quality of life^27^. Authors of an integrative literature review evaluated the determinants of the quality of life of people living with HIV and the most cited variables in the studies are social support, depression, stigma, and adherence to treatment^28^. Another study, whose authors evaluated the quality of life of older people with HIV, observed that the years of living with the disease have a positive influence on quality of life, considering that time is necessary for there to be an adaptation to the changes caused by this condition^29^.

Our results highlight the delimitation of areas with the highest concentration of cases in the metropolitan regions and in Litoral Sul, precisely the most populous in the state. In addition, there is also a concentration of cases in the regions of São José do Rio Preto, Ribeirão Preto, southern Araraquara, and Vale do Paraíba Paulista, which concentrate a large portion of the population in the countryside of the state. These results corroborate other studies whose authors analyzed the distribution pattern of the disease across the country's territories, in which the highest concentration of cases occurs mainly in large urban centers and metropolitan regions, considering the intense flow of people and the concentration of health services^30–33^.

A study on the spatial analysis of factors associated with mortality rates from HIV/AIDS in the older adults in the micro-regions of the south and southeast of Brazil verified that there are regional inequalities in the spatial distribution of mortality^8^. Socioeconomic, demographic, and health factors directly influence these rates, making it necessary to address the social determinants of health when formulating public health policies and healthcare programs focused on HIV/AIDS^30^.

Although associations between spatial results and social determinants of health were not assessed in this study, the literature has shown a tendency towards peripheralization and impoverishment of HIV/AIDS, increasingly representing inequalities in health and disease processes. Authors of a study carried out in the city of São Paulo pointed out clusters of mortality rates from HIV/AIDS in men in places of social vulnerability and showed that, over time, the disease comprised all regions of the city, with a tendency towards peripheralization^34^.

Our study has some limitations, among which stands out the use of information obtained from secondary databases, with the research being subject to underreporting bias or failures in filling out the forms. Another limitation concerns the stigma related to the disease, which may have contributed to the fact that this cause was not mentioned in the death certificates. Nevertheless, the results allowed us characterizing the distribution trend of HIV/AIDS mortality in space over time seeking to indicate the next results and which factors influence this pattern in the older adult population.

All in all, the age group with the highest mortality from HIV/AIDS during the 2010–2020 period was the younger older adults, aged between 60 and 69 years. However, the major growing trend was verified in older people over 69 years of age. During the analyzed period, men were the most prevalent. We observed clusters of lower mortality rates in more central regions of the state, where there is greater urban, population, and economic concentration. Clusters of high mortality rates were located in regions further to the south and north of the state, where places of greater social inequalities are concentrated. In this sense, it is necessary that the actions aimed at HIV/AIDS for this population also consider the geographic contexts, as these are of great importance in the health-disease processes.

## References

[1] Castro SS, Scatena LM, Miranzi A, Miranzi A, Nunes AA (2020). Tendência temporal dos casos de HIV/aids no estado de Minas Gerais, 2007 a 2016. Epidemiol Serv Saúde.

[2] Costa LMCBV, Casseb JSR, Gascon MRP, Fonseca LAM (2018). Características de personalidade e adesão ao tratamento em pacientes jovens portadores de HIV. Rev SBPH.

[3] Kundro MA, Terwel SR, Toibaro JJ, Viloria GA, Losso MH (2016). Late diagnosis of HIV infection in asymptomatic patients. Medicina (B Aires).

[4] Cunha AP, Cruz MM, Pedroso M (2022). Análise da tendência da mortalidade por HIV/AIDS segundo características sociodemográficas no Brasil, 2000 e 2018. Ciên Saúde Coletiva.

[5] Pereira GFM, Shimizu HE, Bermudez XP, Hamann EM (2018). Epidemiologia do HIV e aids no estado do Rio Grande do Sul, 1980-2015. Epidemiol Serv Saúde.

[6] Fenelon MPM, Teixeira MCPA, Silva MV, Lyra BPGS, Amaral LM, Pereira GFM (2021). Epidemiologia of AIDS Brazil, Central-West region and Distrito Federal, 2008-2018. Res Soc Dev.

[7] Silva AQ, Caraciolo JMM, Barbosa MA, Abbate MC, Lopes MEBR, Oliveira ME (2020). Boletim Epidemiológico de IST/AIDS. Cidade de São Paulo [Internet].

[8] Lemes CD, Costa CKF, Gomes CE (2021). Fatores associados à mortalidade por HIV/AIDS em idosos: análise espacial para as microrregiões do Sul e do Sudeste do Brasil. Rev Econ NE.

[9] Silva AT, Parreira ALB, Machado CA, Fonseca DC, Carmo JS, Barbosa ML (2019). Prevalência da AIDS em idosos no centro-oeste brasileiro. REAS/EJCH.

[10] Bhatta M, NandI S, Dutta N, Dutta S, Saha MK (2020). HIV Care among elderly population: systematic review and meta-analysis. AIDS Res Hum Retroviruses.

[11] Pereira RB, Barros CMAR, Silva BBL, Alves AKR, Silva TL (2022). Fatores associados à vulnerabilidade de idosos ao HIV/AIDS: revisão integrativa. Espac Saúde.

[12] Instituto Brasileiro de Geografia e Estatística (2022). Panorama [Internet].

[13] Assunção RM, Barreto SM, Guerra HL, Sakurai E (1998). Mapas de taxas epidemiológicas: uma abordagem Bayesiana. Cad Saúde Pública.

[14] Kim HJ, Fay MP, Feuer EJ, Midthune DN (2000). Permutation tests for joinpoint regression with applications to cancer rates. Stat Med.

[15] Martins-Melo FR, Ramos AN, Alencar CH, Heukelbach J (2016). Trends and spatial patterns of mortality related to neglected tropical diseases in Brazil. Parasite Epidemiol Control.

[16] Anselin L (1995). Local Indicators of Spatial Association-LISA. Geogr Anal.

[17] Lima APR (2020). Sexualidade na terceira idade e HIV. Rev Longeviver.

[18] Aguiar RB, Leal MCC, Marques APO, Torres KMS, Tavares MTDB (2020). Idosos vivendo com HIV – comportamento e conhecimento sobre sexualidade: revisão integrativa. Ciênc Saúde Coletiva.

[19] Rodrigues LR, Portilho P, Tieppo A, Chambo A (2018). Análise do comportamento sexual de idosas atendidas em um ambulatório de ginecologia. Rev Bras Geriatr Gerontol.

[20] Oliveira EL, Neves ALM, Silva IR (2018). Sentidos de sexualidade entre mulheres idosas: relações de gênero, ideologias mecanicistas e subversão. Psicol Soc.

[21] Andrade J, Ayres JA, Alencar RA, Duarte MTC, Parada CMGL (2017). Vulnerabilidade de idosos a infecções sexualmente transmissíveis. Acta Paul Enferm.

[22] Hogan JW, Galai N, Davis WW (2021). Modeling the impact of social determinants of health on HIV. AIDS Behav.

[23] Soares KG, Meneghel SN (2021). O silêncio da sexualidade em idosos dependentes. Ciên Saúde Colet.

[24] Maia DAC, Zani L, Silva ASF, Ambrosano GMB, Flório FM (2018). Notificação de casos de HIV/AIDS em idosos no estado do Ceará: série histórica entre os anos de 2005 a 2014. Rev Bras Geriatr Gerontol.

[25] Vieira CPB, Costa ACSS, Dias MCL, Araújo TME, Galiza FT (2021). Tendência de infecções por HIV/Aids: aspectos da ocorrência em idosos entre 2008 e 2018. Esc Anna Nery.

[26] Santos AABS, Pereira LCS, Pinheiro AS, Barata BQ (2021). Qualidade de vida de idosos vivendo com HIV/AIDS: revisão integrativa. Rev Enferm.

[27] Silva MBG, Santos JAA, Oliveira ESM, Marques KKM, Sales PVO, Costa WC (2020). Qualidade de vida dos portadores de HIV/AIDS no extremo norte do Brasil. REAS/EJCH.

[28] Blandón JAP, Bocanegra AG, Maidana JN, Viana DR, Campos MML (2019). Os determinantes da qualidade de vida em pessoas com HIV: uma revisão integrativa. Rev Enferm UERJ.

[29] Araújo KMST, Leal MCC, Marques APO, Silva SRA, Aguiar RB, Tavares MTDB (2020). Avaliação da qualidade de vida de pessoas idosas com HIV assistidas em serviços de referência. Ciênc Saúde Coletiva.

[30] Paiva SS, Pedrosa NL, Galvão MTG (2019). Análise espacial da AIDS e os determinantes sociais de saúde. Rev Bras Epidemiol.

[31] Maranhão TA, Alencar CH, Ribeiro LM, Sousa GJB, Abreu WC, Pereira MLD (2020). Padrão espaço-temporal da mortalidade por aids. Rev Enferm UFPE On line.

[32] Maranhão TA, Alencar CH, Magalhães MAFM, Sousa GJB, Ribeiro LM, Abreu WC (2020). Mortalidade pela síndrome da imunodeficiência adquirida e fatores sociais associados: uma análise espacial. Rev Bras Enferm.

[33] Limas FM, Brandão ML, Luccas DS, Bossle RC, Khalaf DK, Chaves MMN (2021). Análise especial dos casos de HIV em adultos jovens e o acesso aos serviços públicos em um municipío do Paraná. Mundo da Saúde.

[34] Pellini ACG, Chiaravalloti F, Zanetta DMT (2020). AIDS in men in the city of São Paulo, 1980–2012: spatial and space-time analysis. Rev Saude Publica.

